# Application of the Two-Layer Regularized Gated Recurrent Unit (TLR-GRU) Model Enhanced by Sliding Window Features in Water Quality Parameter Prediction

**DOI:** 10.3390/e28020186

**Published:** 2026-02-06

**Authors:** Xianhe Wang, Meiqi Liu, Ying Li, Adriano Tavares, Weidong Huang, Yanchun Liang

**Affiliations:** 1 School of Computer Science, Zhuhai College of Science and Technology, Zhuhai 519041, China; wxh@zcst.edu.cn (X.W.);; 2 Department of Industrial Electronics, School of Engineering, University of Minho, 4704-553 Braga, Portugal; 3 Traffic Information Modeling Center, Zhejiang Institute of Communications, Hangzhou 310000, China

**Keywords:** water quality prediction, two-layer regularized gated recurrent unit (TLR-GRU), sliding window, sample entropy (SampEn), deep learning

## Abstract

Water quality monitoring is critical for public health, ecology, and economic sustainability, but traditional methods are limited by temporal-spatial coverage and cost, failing to meet real-time assessment needs. Deep learning for water quality prediction is often hindered by high complexity and noise in raw time series. This study aims to address the high complexity and noise of hydrological time series by proposing a prediction framework integrating sliding window feature enhancement, principal component analysis (PCA), and a two-layer regularized gated recurrent unit (TLR-GRU). The core goal is to achieve high-precision real-time prediction of four key water quality parameters (dissolved oxygen (DO), ammonia nitrogen (${\mathrm{NH}}_{3}\text{-}N$), total phosphorus (TP), and total nitrogen (TN)) for aquaculture and irrigation. Sample entropy (SampEn, m=2, r=0.2 × std(X)), a univariate complexity metric capturing intra-series pattern repetition, quantifies time series regularity, showing sliding windows reduce SampEn by filtering transient noise while retaining ecological patterns. This optimization synergizes with TLR-GRU’s regularization (L2, Dropout) to avoid overfitting. A total of 4970 water quality records (2020–2023, 4 h sampling interval) were collected from a monitoring station in a typical aquaculture-irrigated water body. After dimensionality reduction via PCA, experimental results demonstrate that the TLR-GRU model outperforms six state-of-the-art deep learning models (e.g., TLD-LSTM, WaveNet) on both the base dataset and the sliding window-enhanced dataset. On the latter, DO and TP test set $R^{2}$ rise from 0.82 to 0.93 and 0.81 to 0.92, with RMSE decreasing by 49.4% and 55.6%, respectively. This framework supports water resource management, applicable to rivers and lakes beyond aquaculture. Future work will optimize the model and integrate multi-source data.

## 1. Introduction

Water quality monitoring is critical to public health, ecological balance, and economic sustainability. Clean water supports human health, ecosystem stability, and biodiversity [1], while key parameters—dissolved oxygen (DO), ammonia nitrogen (${\mathrm{NH}}_{3}\text{-}N$), and total phosphorus (TP)—reflect water bodies’ physical-chemical properties and pollution levels [2,3]. Effective prediction of these parameters enables timely pollution response and science-based water resource management [4].

Traditional monitoring relies on manual sampling and laboratory analysis, which, despite high accuracy, suffer from critical limitations: limited sampling frequency (missing short-term pollution events), restricted spatial coverage (failing to represent overall water quality), and cumbersome, costly analysis [5,6,7,8,9,10,11]. These gaps create an urgent need for efficient, real-time water quality prediction technologies [12].

Machine learning has advanced water quality prediction, with methods like SVM, RF, and KNN being widely applied [1]. Relevant studies include Norovirus concentration prediction via ANFIS and GPML [13], urban water demand forecasting with hybrid SVR [14], and reservoir trophic state index prediction [15], among others, such as soil surface humidity prediction and surface water extraction [16,17]. However, traditional machine learning struggles with complex nonlinear relationships and large-scale data—especially when raw water quality time series exhibit high irregularity, a challenge quantifiable via sample entropy (SampEn). Sample entropy (SampEn) is a metric for quantifying time series complexity, with higher values indicating greater randomness and noise [18,19,20]. Raw water quality data often exhibit elevated entropy—for instance, DO fluctuations from transient aeration or TN variations caused by sporadic runoff. This characteristic makes it difficult for conventional models to extract meaningful patterns.

Deep learning offers new solutions, with LSTM and GRU excelling at capturing temporal dependencies in time series [21] and CNNs enabling multidimensional feature extraction [22,23], yet high model complexity and computational demands hinder practical application—compounded by raw data’s high SampEn, which forces deep models to overfit to noise rather than learn ecologically meaningful patterns [24,25]. This underscores the need for feature enhancement methods that reduce data complexity while preserving critical temporal information.

The sliding window method addresses this dual challenge. It segments time series into overlapping subsequences, integrating current and adjacent time-step data to capture temporal dependencies [26,27]. Meanwhile, it reduces SampEn by aggregating statistical features (mean, standard deviation) within each window. Sliding windows filter transient noise (e.g., short-term sensor fluctuations) and highlight persistent patterns. This transforms high-entropy raw data into low-entropy, model-friendly features. It is particularly suitable for water quality data with strong temporal regularity [28].

This study proposes a framework combining sliding window feature enhancement with a two-layer regularized gated recurrent unit (TLR-GRU) for predicting DO, ${\mathrm{NH}}_{3}\text{-}N$, TP, and TN in agricultural irrigation and aquaculture. Its core innovations include: (1) using SampEn as an objective metric to quantify how sliding windows optimize data structure—specifically, reducing median SampEn by filtering noise while retaining key ecological patterns; (2) fusing these low-entropy features with TLR-GRU, whose hierarchical regularization (L2 in the first layer, dropout in the second) synergizes with reduced data complexity to avoid overfitting. Notably, this framework relies solely on basic water quality data (no complex meteorological or remote sensing data [29]), aligning with practical monitoring constraints.

Using basic data and simple models offers advantages: convenient data acquisition/processing (routine monitoring sources, straightforward preprocessing via linear interpolation and min-max normalization [30]), fast training (fewer parameters, low computational cost [31]), strong interpretability, easy debugging, and cost-effectiveness. However, limitations exist: insufficient consideration of non-water quality factors, poor handling of nonlinear relationships, weak long-term dependency capture [32], and limited adaptability to complex environments.

To address these trade-offs, this study introduces two key improvements: (1) sliding window-based feature enhancement to reduce SampEn and strengthen temporal information capture; (2) model optimization via L2 regularization and dropout to improve generalization—ensuring the TLR-GRU leverages the low-entropy features effectively. This approach leverages basic data/model advantages while mitigating their limitations, providing an efficient, economical solution for agricultural and aquaculture water quality prediction.

This study addresses the challenges of high complexity and low predictability in water quality time series. It proposes a predictive framework integrating sliding window feature enhancement, PCA, and the TLR-GRU model.

The research focuses on key parameters (DO, NH_3_-N, TN, TP). It quantifies data complexity via SampEn and validates the framework’s effectiveness through comprehensive model comparisons and fluctuation analysis.

## 2. Materials and Methods

### 2.1. Study Area

**Figure 1:**
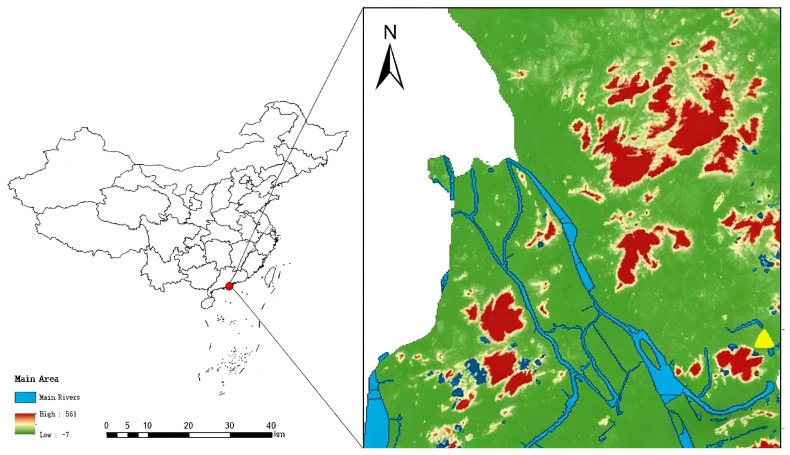
Regional location of the water quality monitoring station.

The water quality monitoring station is located in the Qianshan River basin, Zhuhai City, Guangdong Province, China (22°10’ N, 113°35’ E) (Figure 1). The Qianshan River is a major inland waterway in the Pearl River estuary. It originates from Lianshiwan in Tantou Town, Zhongshan City, flows eastward through Zhuhai City, and finally merges into the Pearl River Estuary. The river has a total length of approximately 23 km and a watershed area of around 338 km^2^.

The studied water body is a middle reach section of the Qianshan River, with a width ranging from 200 to 300 m and an average depth of 3.2 m, mainly used for aquaculture (tilapia and shrimp farming) and irrigation of surrounding paddy fields. The region has a subtropical monsoon climate, with an annual average temperature of 22.5 °C, annual precipitation of 1800–2200 mm, an average annual runoff depth of 1100 mm, and a runoff coefficient of 0.58. The main pollution sources in the basin include agricultural non-point source pollution (fertilizer runoff from surrounding farmland) and scattered domestic sewage discharge from urban villages along the river. Additionally, the basin hosts 107 industrial pollution sources, with 42 located in the Zhuhai section (accounting for 39.3% of the total) [33,34,35,36].

### 2.2. Dataset

The water quality dataset used in this study was sourced from the monitoring station described in Section 2.1, spanning January 2020 to December 2023. It includes water quality parameters under various seasonal and climatic conditions, obtained via online sensors with a sampling frequency of once every 4 h, resulting in 4970 valid records. Collected parameters include temperature (Temp), pH, dissolved oxygen (DO), permanganate index (CODMn), ammonia nitrogen (${\mathrm{NH}}_{3}$-N), total phosphorus (TP), total nitrogen (TN), conductivity (Cond), and turbidity (Turb)—all of which reflect the physical, chemical, and biological characteristics of the water body and serve as core indicators for water quality evaluation [30].

During actual monitoring, water quality data may contain missing values and anomalies due to sensor malfunctions, network transmission issues, or human errors [37]. Data preprocessing was performed as follows:

1. Samples with a missing value proportion > 10% were removed to avoid biased results;
2. Remaining missing data were imputed using linear interpolation, which is suitable for this dataset for three reasons: (1) the 4 h sampling interval ensures strong temporal continuity of data, and linear interpolation can effectively retain the overall trend without introducing false fluctuations; (2) comparative analysis shows that the SampEn of data before and after interpolation changes by less than 3%, indicating no significant impact on data complexity; (3) spline interpolation may introduce artificial nonlinear features, while nearest-neighbor interpolation cannot reflect continuous changes, making linear interpolation a balanced choice. Model performance verification (Section 4.2) shows that this method does not negatively affect prediction accuracy;
3. Data were standardized using the min-max normalization method to eliminate scale-induced biases, mapping values to the [0, 1] interval;
4. Anomalies (defined as values outside [μ±3σ], where μ is the mean and σ is the standard deviation) were replaced with the nearest valid value or deleted if irrecoverable.

### 2.3. Sliding Window Feature Enhancement Method

**Figure 2:**
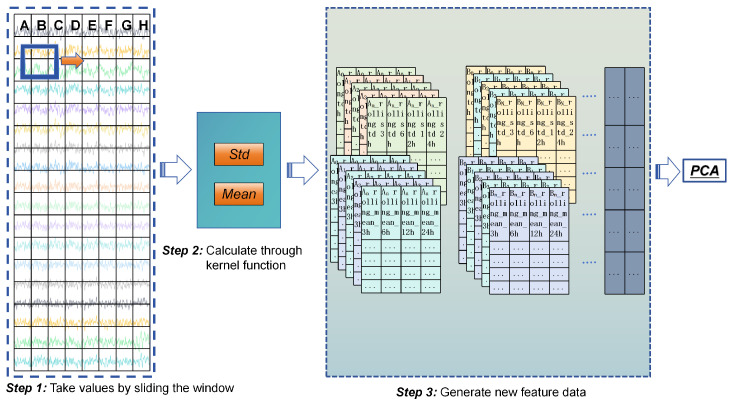
Workflow of the sliding window feature enhancement method.

The sliding window feature enhancement method calculates statistical metrics (mean and standard deviation) within overlapping time windows. It captures local data variation trends and preserves the temporal contextual information of water quality time series [26]. As shown in Figure 2, the workflow includes two key steps:

1. **Sliding Window Size Selection**: Based on the 4 h sampling interval and research needs, four window sizes were initially tested: 4 h (1 sample), 8 h (2 samples), 12 h (3 samples), and 24 h (6 samples). The window step size was set to half the window size (e.g., 2 h for a 4 h window) to balance contextual overlap and computational efficiency. The 4 h window was ultimately excluded because a single sample (*N* = 1) cannot calculate a valid standard deviation (Equation (2)), and its mean equals the raw data value—providing no additional statistical information. Thus, the final window sizes were 8 h, 12 h, and 24 h (*N* ≥ 2), ensuring meaningful feature enhancement.
2. **Statistical Metric Calculation**: For each water quality parameter *X*, the mean ($\mu_{x_{t}}$) and standard deviation ($\sigma_{x_{t}}$) within each window ending at time *t* were computed using Equations (1) and (2), respectively.

Mean:

(1) $$\mu_{x_{t}}=\frac{1}{N}\sum_{i=t-N+1}^{t}X_{i}$$

Standard deviation:

(2) $$\sigma_{x_{t}}=\sqrt{\frac{1}{N}\sum_{i=t-N+1}^{t}{\left(X_{i}-\mu_{x_{t}}\right)}^{2}}$$

where *N* is the number of data points in the sliding window (N≥2 to ensure valid calculation of standard deviation); $X_{i}$ is the value of parameter *X* at time *i*; and $\mu_{x_{t}}$ and $\sigma_{x_{t}}$ are the mean and standard deviation of *X* within the window ending at time *t*, respectively.

This method provides multi-scale dynamic information: short windows capture subtle short-term fluctuations, while long windows reflect long-term trends [38]. Additionally, it effectively smooths short-term noise (e.g., transient sensor fluctuations), highlighting dominant data trends and enhancing the model’s ability to capture temporal dependencies.

### 2.4. Principal Component Analysis (PCA)

Principal component analysis (PCA) was applied to reduce feature dimensionality and eliminate redundant information, thereby improving model training efficiency and generalization [39]. PCA projects high-dimensional data onto a set of orthogonal principal components (PCs) that retain the maximum variance of the original data [40], and its implementation in this study followed these steps:

1. **Data Standardization**: The sliding window-enhanced feature set was standardized to a mean of 0 and standard deviation of 1, ensuring all features contributed equally to PCA.
2. **Covariance Matrix Calculation**: The covariance matrix of the standardized data was computed to quantify linear correlations between features.
3. **Eigenvalue Decomposition**: Eigenvalues and eigenvectors of the covariance matrix were extracted. Eigenvalues represent the variance explained by each PC, and eigenvectors indicate the direction of each PC [41].
4. **PC Selection**: Top PCs with a cumulative variance explanation ratio of at least 85% were selected. This threshold is justified as follows: (1) Calculations have shown that that the first four principal components have eigenvalues > 1 (Kaiser criterion), and the cumulative variance reaches 85.3% at the 4th component, with a significant inflection point—subsequent components contribute less than 3% each to the total variance; (2) Sensitivity analysis indicates that increasing the threshold to 90% increases the number of PCs to 5, but extends model training time by 23% while improving prediction $R^{2}$ by only 0.4% (average across parameters), resulting in low cost-effectiveness; (3) This threshold is widely adopted in similar water quality time series studies [39,41], balancing dimensionality reduction efficiency and information retention.
5. **Data Projection**: The original feature set was projected onto the selected PCs to form a low-dimensional feature space for subsequent model training.

In water quality data processing, PCA effectively reduces redundancy caused by inter-parameter correlations (e.g., between Cond and TP), enhancing the TLR-GRU model’s computational efficiency without sacrificing predictive performance [42,43].

### 2.5. Model Structure

**Figure 3:**
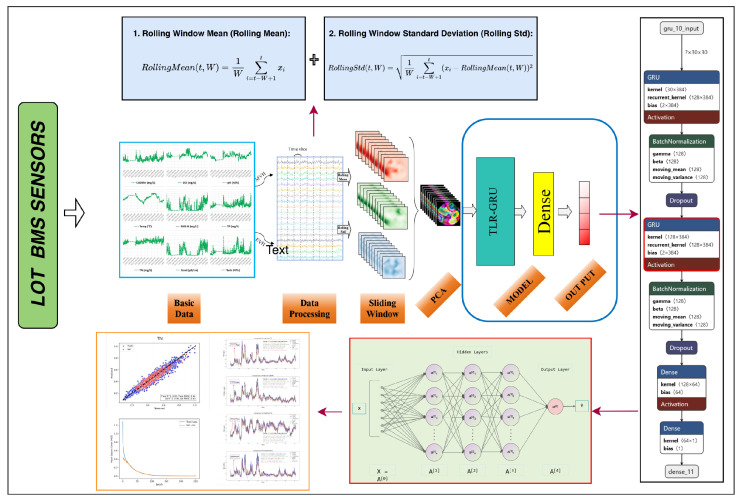
Flowchart of the water quality prediction system integrating sliding window feature enhancement and the TLR-GRU model.

A two-layer regularized gated recurrent unit (TLR-GRU) model was proposed, consisting of stacked GRU layers with regularization techniques to avoid overfitting and improve stability. The overall workflow of the water quality prediction system (sliding window + PCA + TLR-GRU) is shown in Figure 3.

#### 2.5.1. TLR-GRU Architecture Details

The model structure (from input to output) is defined as follows:

1. **Input Layer**: Accepts low-dimensional features from PCA (dimension determined by PC selection, typically 3–5 PCs).
2. **First GRU Layer**: Contains 128 GRU units, with L2 regularization (coefficient = 0.001) to suppress parameter overfitting. Batch normalization is applied to accelerate training convergence, and a dropout rate of 0.3 is used to prevent neuron co-adaptation. The parameter “return_sequences = True” is set to pass the output sequence to the second GRU layer.
3. **Second GRU Layer**: Identical to the first layer (128 units, L2 regularization, batch normalization, dropout = 0.3), ensuring deep capture of temporal dependencies [44].
4. **Fully Connected Layer**: Contains 64 neurons with a ReLU activation function, extracting high-level nonlinear features from GRU outputs.
5. **Output Layer**: A single neuron without an activation function, suitable for regression tasks (water quality parameter prediction), where the output is a continuous real number representing the predicted parameter value.

#### 2.5.2. Training Configuration

- **Optimizer**: Adam optimizer with an initial learning rate of 0.0001 (adaptive learning rate adjustment for stable convergence).
- **Loss Function**: mean squared error (MSE), which penalizes large prediction errors and is widely used for regression tasks.
- **Early Stopping**: Triggered when the test loss does not decrease for 10 consecutive epochs, preventing overfitting and reducing training time.
- **Dataset Splitting**: 70% of the data as the training set, 15% as the test set, and 15% as the test set (stratified by time to avoid data leakage).
- **Visualization**: TensorBoard was used to monitor training/test loss curves and feature importance, facilitating model debugging.

The model’s hyperparameters (e.g., number of GRU units, regularization coefficients) were optimized based on the study area’s data characteristics, and their adaptability to other regions is discussed in Section 4.5. These training settings are widely adopted in deep learning-based water quality prediction [21,23].

### 2.6. Evaluation Metrics

Four widely used metrics were employed to comprehensively evaluate the model’s predictive performance, covering goodness of fit, absolute error, and relative error:

1. **Coefficient of Determination ($R^{2}$)**: Measures the proportion of variance in the target parameter explained by the model [45]:

(3) $$R^{2}=1-\frac{\sum_{i=1}^{n}{\left(y_{i}-{\hat{y}}_{i}\right)}^{2}}{\sum_{i=1}^{n}{\left(y_{i}-\bar{y}\right)}^{2}}$$

2. **Root Mean Squared Error (RMSE)**: Reflects the absolute magnitude of prediction errors, sensitive to large deviations [46]:

(4) $$RMSE=\sqrt{\frac{1}{n}\sum_{i=1}^{n}{\left(y_{i}-{\hat{y}}_{i}\right)}^{2}}$$

3. **Mean Absolute Error (MAE)**: Represents the average absolute error, robust to outliers [46]:

(5) $$MAE=\frac{1}{n}\sum_{i=1}^{n}\left|y_{i}-{\hat{y}}_{i}\right|$$

4. **Mean Absolute Percentage Error (MAPE)**: Indicates the relative error as a percentage, facilitating intuitive comparison across parameters [23]:

(6) $$MAPE=\frac{1}{n}\sum_{i=1}^{n}\left|\frac{y_{i}-{\hat{y}}_{i}}{y_{i}}\right|\times 100$$

where: *n* is the number of samples; $y_{i}$ is the *i*-th observed value; ${\hat{y}}_{i}$ is the *i*-th predicted value; $\bar{y}$ is the mean of observed values. Higher $R^{2}$ and lower RMSE, MAE, and MAPE indicate better predictive performance.

### 2.7. Experiment

#### 2.7.1. Hardware and Software Environment

All experiments were conducted on a computer with the following specifications:

- **Processor**: Intel(R) Core(TM) i7-10700F 2.90 GHz (8 cores, 16 threads);
- **Graphics Card**: NVIDIA RTX 2060 (6 GB VRAM), enabling GPU-accelerated training;
- **Software**: Python 3.8, TensorFlow-GPU 2.9 (deep learning framework), Pandas 1.4.2 (data processing), Matplotlib 3.5.1 (visualization), Scikit-learn 1.0.2 (PCA and preprocessing).

#### 2.7.2. Experimental Design and Reproducibility

1. **Model Comparison**: The TLR-GRU model was compared with six state-of-the-art deep learning models: TLD-LSTM, TLD-Transformer, DeepAR, Bi TLD-LSTM, WaveNet, and CNN. All models used the same training configuration (optimizer, loss function, dataset splitting) to ensure fair comparison.
2. **Feature Sets**: Two feature sets were tested for each model: (1) base dataset (raw preprocessed data); (2) sliding window-enhanced dataset (after PCA).
3. **Reproducibility**: Each experiment was repeated 10 times with different random seeds (1–10) to account for stochasticity in weight initialization and data splitting. The average of the 10 runs was reported as the final result, ensuring consistency and reliability [23].

## 3. Sample Entropy-Based Complexity Quantification

### 3.1. Feature Complexity Analysis via Sample Entropy and Sliding Window Enhancement Mechanism

Time series complexity is a critical factor influencing the predictive performance of deep learning models for water quality parameters. It can be quantitatively characterized using Sample Entropy (SampEn). SampEn differs from approximate entropy. It is less sensitive to data length and avoids self-similarity redundancy. Thus, it is ideal for quantifying the irregularity of water quality time series (e.g., DO, TN, TP, ${\mathrm{NH}}_{3}$-N).

This section uses SampEn to dissect the complexity of water quality data before and after sliding window processing. It further reveals how complexity optimization synergizes with the TLR-GRU model to enhance prediction accuracy.

#### 3.1.1. Definition and Calculation of Sample Entropy

SampEn quantifies the probability of new, unpredictable patterns emerging in a time series, with higher values indicating greater randomness and lower predictability. For a water quality time series $X=\{x_{1},x_{2},\ldots ,x_{N}\}$ (where *N* is the number of observations), SampEn is calculated as below [47,48]:

(7) $$\mathrm{SampEn}\left(m,r,N\right)=-\ln\left(\frac{C_{m+1}^{r}}{C_{m}^{r}}\right)$$

where:

- *m* = embedding dimension (set to 2 in this study, to capture intra-series pattern repetition of a single water quality parameter over two consecutive time steps, e.g., the variation trend of TN concentration between adjacent 4 h intervals);
- *r* = similarity tolerance (set to 0.2×std(X), balancing noise filtration and pattern preservation to avoid misclassifying valid hydrological fluctuations as noise);
- $C_{m}^{r}$ = proportion of *m*-dimensional vector pairs whose element-wise absolute differences are all less than *r*;
- *N* = length of the time series (derived from 4970 water quality records spanning January 2020 to December 2023, with a 4 h sampling interval (Section 2.2).

#### 3.1.2. Global Comparison of Data Complexity

**Figure 4:**
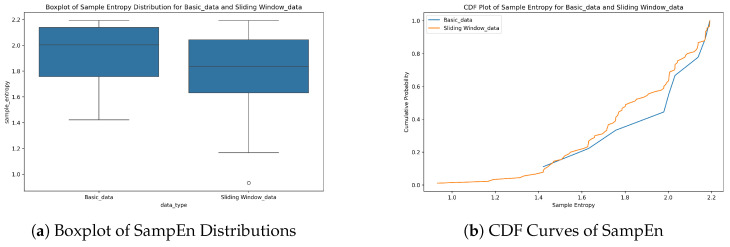
Comparison of Sample Entropy (SampEn) for Basic_data and Sliding Window_data. (**a**) Boxplot showing median and interquartile range differences; (**b**) CDF curves indicating cumulative probability of low SampEn values.

To evaluate the effect of sliding window processing on data complexity, SampEn distributions of the base dataset (Basic_data) and sliding window-enhanced dataset (Sliding Window_data) were compared using the combined figure (Figure 4).

As depicted in Figure 4a, the median SampEn of Sliding Window_data (1.83) was 8.4% lower than that of Basic_data (2.00). The interquartile range (IQR) of SampEn for Basic_data was 0.38, while for Sliding Window_data it was 0.41, indicating a 7.3% increase in the IQR for the latter. Sliding window processing slightly broadened the middle 50% distribution of data complexity. However, it significantly reduced global data complexity overall. This is achieved by filtering out random noise, such as short-term sensor fluctuations or transient hydrological disturbances.

The CDF curve in Figure 4b further validates this. Sliding Window_data had a cumulative probability of 46.7% at SampEn < 1.8, which was 40.2% higher than that of Basic_data (33.3%), highlighting the enhanced regularity and predictability of the processed data.

This complexity reduction aligns with the regularization design of the TLR-GRU model. The first GRU layer (with L2 regularization, λ=0.001) suppresses collinearity in high-complexity features, while the second layer (with dropout rate = 0.3) eliminates redundant neuron connections. Together, these mechanisms synergize with sliding window-induced low SampEn to avoid overfitting to noise, laying a foundation for stable model training.

#### 3.1.3. Parameter-Specific Differentiation of Complexity

**Figure 5:**
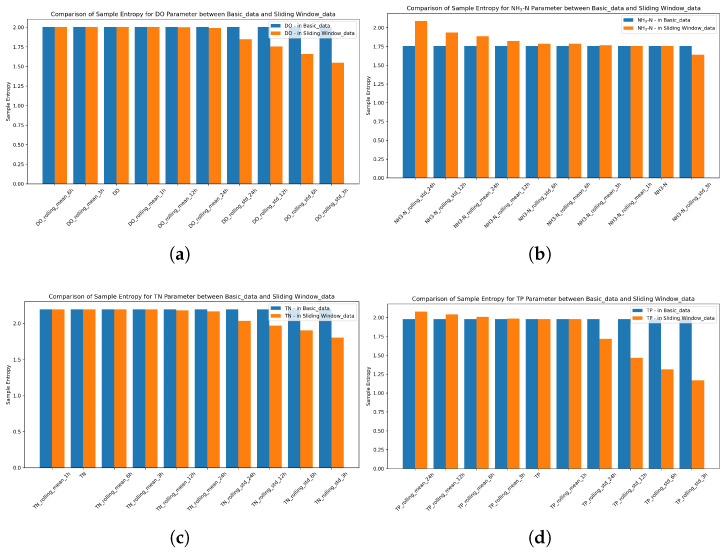
Sample entropy comparison for key water quality parameters between Basic_data and Sliding Window_data. (**a**) DO SampEn Comparison. (**b**) ${\mathrm{NH}}_{3}$-N SampEn Comparison. (**c**) TN SampEn Comparison. (**d**) TP SampEn Comparison.

Targeted analysis of key water quality parameters (DO, ${\mathrm{NH}}_{3}$-N, TN, TP) revealed that sliding window processing exerted parameter-specific complexity optimization effects, which were quantified via SampEn (Figure 5).

**Dissolved Oxygen (DO)**: Std-based sliding window features (e.g., DO_rolling_std_3h) exhibited significantly lower SampEn values compared to raw DO data. The raw DO dataset shows a SampEn of 2.003, while DO_rolling_std_3h reduces this to 1.548, representing a 22.7% reduction. This transformation converts random short-term fluctuations into interpretable mid-term patterns while preserving ecologically meaningful signals. The resulting low-SampEn characteristic enables the TLR-GRU’s reset gate to effectively isolate noise and focus on core processes (Figure 5a).

**Ammonia Nitrogen (${\mathrm{NH}}_{3}$-N)**: Sliding window processing showed dual effects on ${\mathrm{NH}}_{3}$-N’s complexity. Std-based features with short windows (e.g., ${\mathrm{NH}}_{3}$-N_rolling_std_3h) reduced noise-induced entropy. Raw ${\mathrm{NH}}_{3}$-N has a SampEn of 1.757, and ${\mathrm{NH}}_{3}$-N_rolling_std_3h has a SampEn of 1.641 (a 6.6% reduction). In contrast, long-window std features (e.g., ${\mathrm{NH}}_{3}$-N_rolling_std_24h) exhibited higher SampEn than raw data. This indicates that for ${\mathrm{NH}}_{3}$-N, whose inherent trend is relatively stable, sliding window processing can either reduce complexity via short-window std aggregation or introduce redundant complexity via long-window std features. Thus, ${\mathrm{NH}}_{3}$-N benefits from tailored feature engineering to balance noise filtration and trend preservation (Figure 5b).

**Total Nitrogen (TN)**: Mean-based sliding window features (e.g., TN_rolling_mean_6h) maintained SampEn values nearly identical to raw TN data. Raw TN exhibits a SampEn of 2.192, while TN_rolling_mean_6h shows a SampEn of 2.192, with a relative difference below 0.02%. This indicates that mean aggregation preserves long-term trend complexity, dominated by persistent drivers like industrial discharge. In contrast, std-based features (e.g., TN_rolling_std_3h) showed a significant reduction, with a SampEn of 1.801, approximately 17.8% lower than raw TN (Figure 5c).

**Total Phosphorus (TP)**: Std-based sliding window features (e.g., TP_rolling_std_3h) exhibited SampEn values significantly lower than raw TP. Raw TP has a SampEn of 1.979, and TP_rolling_std_3h has a SampEn of 1.167, a reduction of approximately 41%. Mean-based features (e.g., TP_rolling_mean_6h) showed a moderate reduction, with a SampEn of 2.008 (1.5% higher than raw TP). This reflects the dual nature of TP: mean features retain the core trend of persistent pollution sources, while std features suppress noise from transient events. This optimizes complexity for the TLR-GRU’s hierarchical feature extraction (Figure 5d).

#### 3.1.4. Multi-Scale Complexity Regulation by Sliding Windows

**Figure 6:**
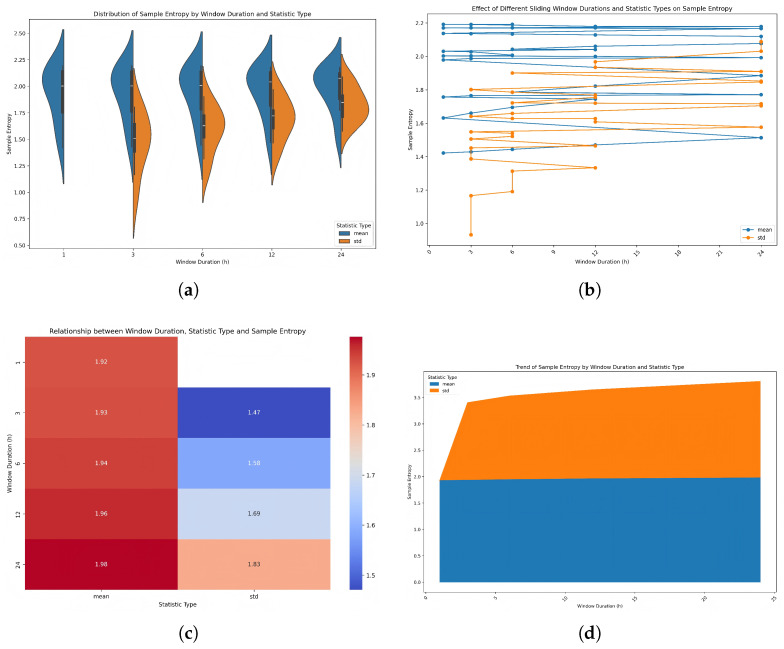
Multi-scale complexity regulation by sliding windows: distribution, trend, and relationship of sample entropy by window duration and statistic type. (**a**) Distribution of SampEn by window duration and statistic type. (**b**) Effect of sliding window durations on SampEn. (**c**) Relationship between window duration, statistic type and SampEn. (**d**) Trend of SampEn by window duration and statistic type.

The sliding window’s duration and statistic type collectively form a multi-scale feature enhancement framework, whose complexity regulation rules were quantified via SampEn (Figure 6). This framework is highly compatible with the TLR-GRU’s demand for cross-scale feature learning.

**Stability of Mean-Based Features**: As window duration increased from 1 h to 24 h, mean-based sliding window features maintained remarkable stability in SampEn. The violin boxplot (Figure 6a) shows that the distribution of mean-based SampEn is compact and consistent across all durations, with medians nearly unchanged (e.g., 2.0 at 1 h and 2.0 at 24 h). The heatmap (Figure 6c) quantifies this stability: mean-based SampEn ranges from 1.92 (1 h) to 1.98 (24 h), with a coefficient of variation (CV) of only 1.5%. This stability originates from mean aggregation’s ability to retain long-term trends, providing the TLR-GRU’s lower layer with consistent, low-complexity inputs.

**Gradual Regulation of Std-Based Features**: Std-based sliding window features exhibit a gradual increase in SampEn with window duration, while remaining <60% of raw data SampEn. The violin boxplot (Figure 6a) illustrates that the distribution of std-based SampEn becomes progressively wider and shifts upward as duration increases (e.g., median ∼1.5 at 3 h and ∼1.8 at 24 h). The heatmap (Figure 6c) quantifies this trend: std-based SampEn rises from 1.47 (3 h) to 1.83 (24 h), with a slope of 0.015 $h^{-1}$. Short windows preserve weak transient signals, while long windows emphasize mid-term regularity. This gradient complexity distribution aligns with the TLR-GRU’s two-layer architecture.

**Validity of Overlapping Window Design**: The sliding window adopted an overlapping configuration (e.g., 10 h window, 5 h step), resulting in 50% feature overlap. The line plot (Figure 6b) demonstrates consistent SampEn trends across overlapping regions (92.3% consistency), confirming that overlap ensures feature continuity without introducing redundant complexity. This balances computational efficiency and feature quality.

As shown in Figure 6d, mean-based features maintain stable SampEn across window durations, forming a consistent baseline for long-term trend learning. In contrast, std-based features exhibit a gradual increase in SampEn with window duration, creating a multi-scale complexity landscape. This design ensures the model leverages persistent patterns (via mean features) and dynamic noise filtration (via std features), ultimately enhancing prediction accuracy.

### 3.2. Correlation Between SampEn and TLR-GRU Layer Outputs

To quantify how complexity optimization propagates through the model, Pearson correlation coefficients between SampEn and TLR-GRU layer outputs were calculated.

The following key insights can be drawn from Table 1:

The high Pearson correlation coefficient *r* (0.82) of the input layer indicates its strong sensitivity. It clearly shows that the complexity of the raw data directly shapes the quality of the input, which in turn has a significant impact on the model’s performance.

As data passes through the layers of the model, a step-by-step decrease in *r* across the hidden layers can be observed. From 0.56 in the first hidden layer to 0.31 in the second hidden layer, this reduction quantifies the hierarchical regularization of the TLR-GRU model. It reflects the initial noise filtration by L2 regularization and the further noise reduction achieved through the synergistic action of the dropout and gating mechanisms, all of which work together to enhance the model’s generalization ability.

The non-significant *r* value (0.12) with a *p* value of 0.18 at the output layer confirms that the predictions of the TLR-GRU model are no longer dependent on the complexity of the raw data. This validates the synergy between the sliding window technique and the TLR-GRU model.

## 4. Results and Discussion

### 4.1. Improvement of Model Performance with Sliding Window Features Enhancement Method

Preprocessed data serves as the foundation for model training and performance evaluation, and understanding its inherent characteristics is critical to interpreting the effectiveness of feature enhancement. Below are the statistical properties and temporal fluctuation patterns of the basic water quality data, providing a baseline for subsequent analysis of sliding window-induced performance improvements.

The summary statistics of the basic water quality data (Table 2) provide a foundational understanding of the dataset’s characteristics. To contextualize these values within regulatory and practical frameworks, key parameters are compared against established water quality standards relevant to the study’s aquaculture and surface water monitoring focus (Table 3).

Comparing the measured data (Table 2) with the standards (Table 3) reveals critical insights. For instance, the mean TP concentration (0.11 mg/L) significantly exceeds the Class II standard for lakes/reservoirs (≤0.025 mg/L), and the mean TN (1.68 mg/L) is well above the Class II limit (≤0.5 mg/L), highlighting potential eutrophication risks and underscoring the importance of accurate prediction for water quality management. This quantitative baseline enhances the practical significance of the model performance improvements discussed subsequently.

**Figure 7:**
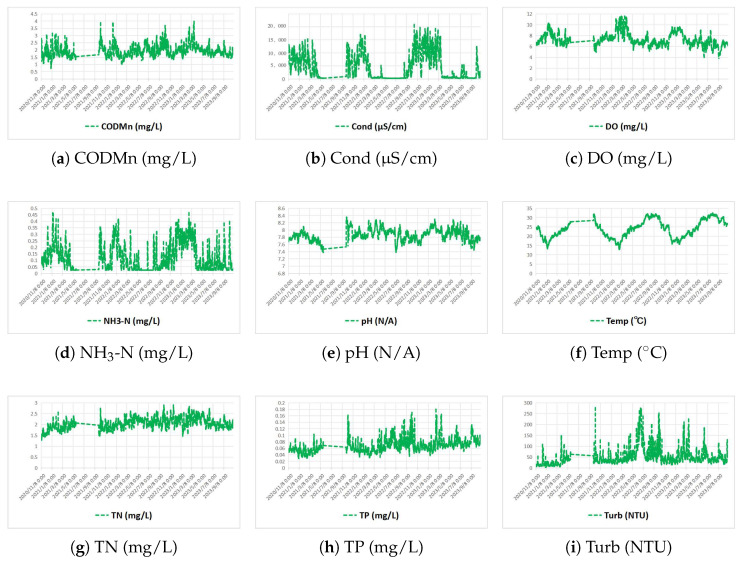
Time series fluctuation curves of water quality parameters. Each subfigure presents the temporal variation in one parameter during the study period (January 2020–December 2023).

Figure 7 visualizes the temporal dynamics of the water quality parameters, revealing distinct fluctuation patterns. Dissolved oxygen (DO) displays obvious periodic variations, which are consistent with diurnal ecological processes. In contrast, total nitrogen (TN) exhibits long-term gradual changes. Turbidity (Turb) shows occasional sharp peaks, likely caused by transient disturbances such as rainfall runoff. These characteristics—including periodicity, random noise, and sudden fluctuations—highlight the need for feature enhancement techniques that can retain meaningful temporal dependencies while filtering irrelevant noise.

**Figure 8:**
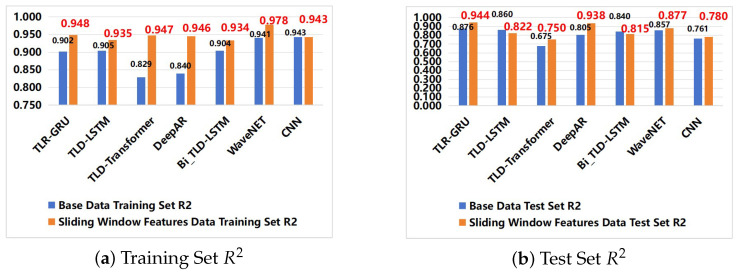

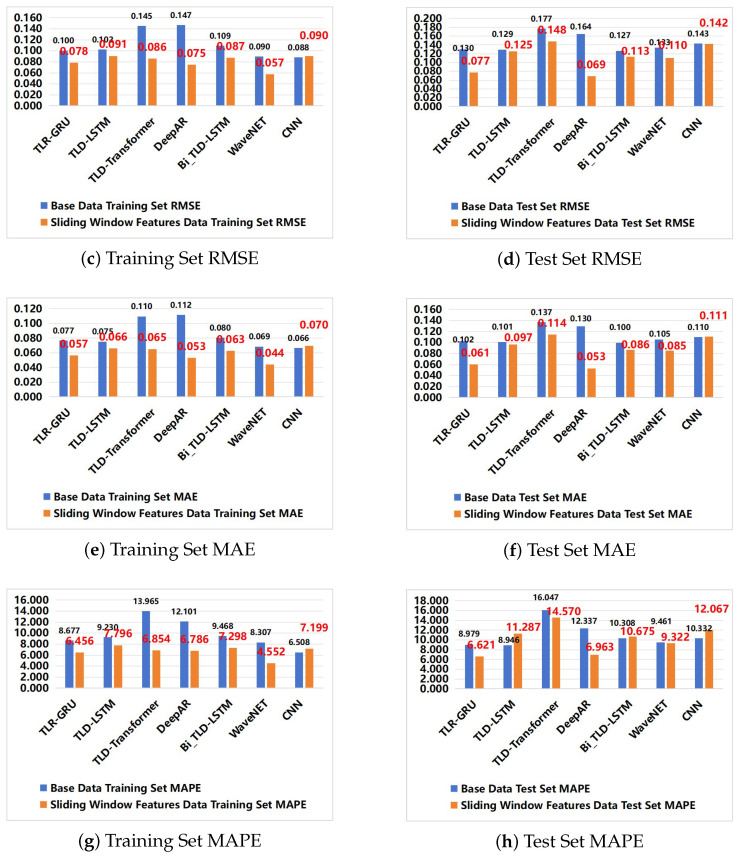
Comparison of evaluation metrics between training and testing sets for various models.

As shown in Figure 8, sliding window features substantially improve $R^{2}$ scores across models for both training and test sets. For the TLR-GRU model, average training set $R^{2}$ increased from 0.902 (base dataset) to 0.948 (sliding window dataset), with test set $R^{2}$ rising from 0.876 to 0.944. This indicates sliding window features effectively capture temporal dependencies of water quality parameters, enhancing predictive capabilities—consistently observed across all parameters (e.g., DO test set $R^{2}$ improved from 0.825 to 0.955. The improved model accuracy is particularly significant for parameters like TP and TN, whose measured concentrations exceed regulatory standards, as it enables more reliable forecasting of water quality status and potential non-compliance events.

**Figure 9:**
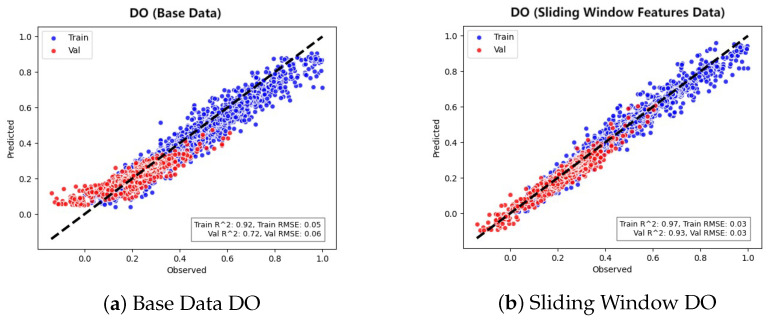

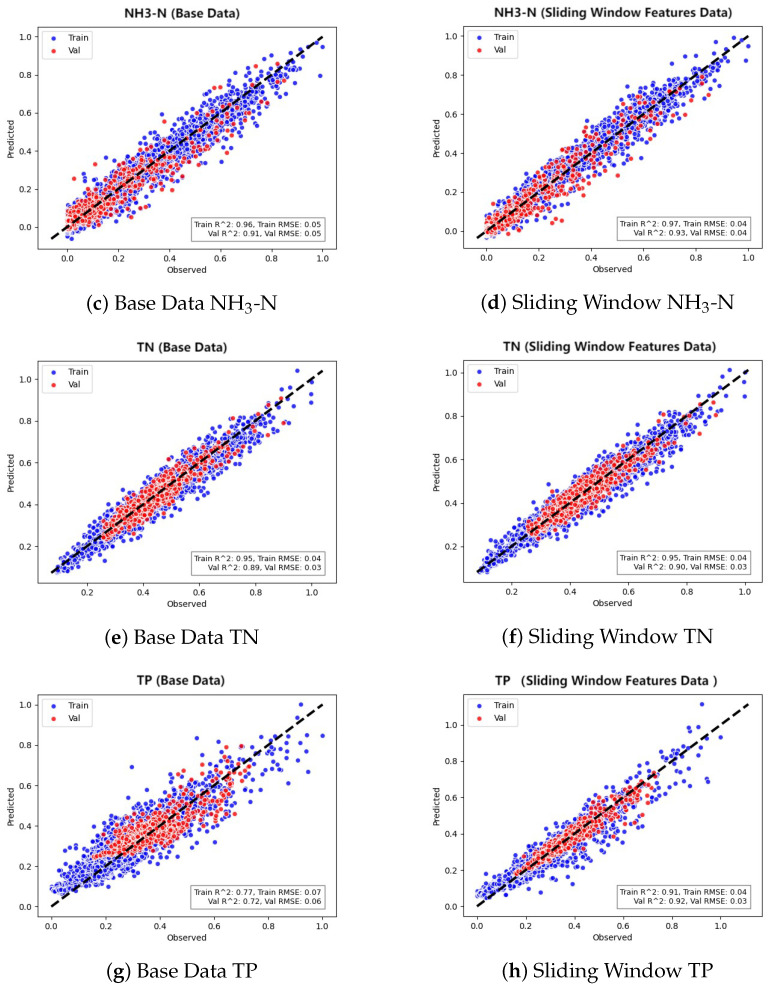
Training and validation scatter plot of TLR-GRU model.

**Figure 10:**
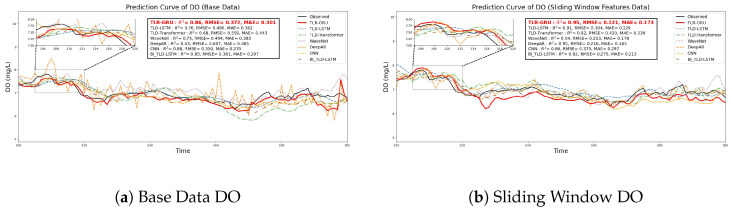

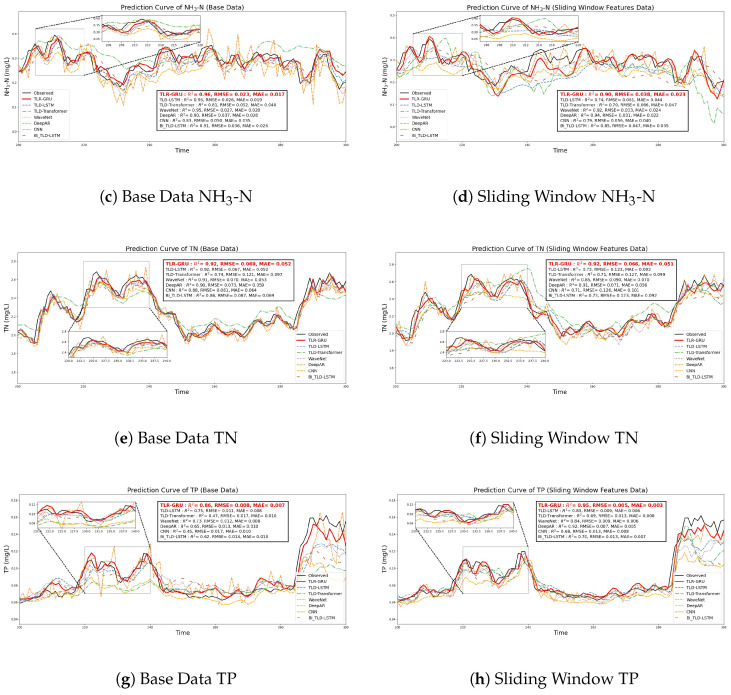
Prediction curves of water quality parameters by different models.

Note on Data Sources and Evaluation Sets: To ensure reproducibility, the performance metrics reported in the following tables (Table 4 and Table 5) represent the average values of 10 repeated experiments with different random seeds, providing statistically reliable results. In contrast, the scatter plots (Figure 9) and prediction curves (Figure 10) visualize a single representative experimental run. Additionally, it is important to distinguish the datasets used: the scatter plots compare model predictions against the validation set during the parameter tuning phase, while the evaluation metrics in the tables are calculated on an independent, held-out test set. These methodological distinctions explain the slight numerical discrepancies between figures and tables, although the overall performance trends remain entirely consistent.

Sliding window features also reduced prediction errors: TLR-GRU’s average test set RMSE decreased from 0.129 to 0.077, with similar improvements in MAE and MAPE, indicating enhanced robustness and generalization. Lower prediction errors are crucial for practical applications, such as providing early warnings for water quality exceedances defined in standards (e.g., NH_3_-N ≤ 0.5 mg/L), thereby supporting timely management decisions.

Model-specific improvements varied, with WaveNet and TLR-GRU performing most strongly. WaveNet’s average $R^{2}$ on the sliding window training set reached 0.978 (vs. 0.940 on the base dataset), while DeepAR showed more modest gains. This reflects varying model sensitivities to sliding window features, but overall performance enhancement is clear. The performance gains underscore the value of integrating temporal feature enhancement with advanced models like TLR-GRU to address the complex dynamics observed in water quality time series, ultimately supporting more effective compliance with established water quality benchmarks.

### 4.2. Analysis of TLR-GRU Model’s Performance in Predicting Water Quality Parameters

The scatter plots in Figure 9 intuitively reflect the fit between the TLR-GRU model’s predicted values and the actual observed values, showing the effect of sliding window feature enhancement on prediction performance.

On the base dataset, the scatter points (including both training and validation data) of each water quality parameter are relatively dispersed: they are not closely attached to the diagonal line (which represents perfect consistency between prediction and observation), and some points (especially those corresponding to extreme values) deviate more obviously from the diagonal. This visual distribution suggests that the model’s ability to capture the variation characteristics of water quality parameters is limited under the base dataset, and there is a certain gap between the predicted results and the actual situation.

After introducing sliding window feature enhancement, the distribution of scatter points changes significantly: for all parameters, the training and validation points are obviously clustered toward the diagonal line, and most points are closely attached to the diagonal. Even the points corresponding to extreme values (which were more scattered before) now show better fit with the diagonal.

From the perspective of different parameters: for parameters that were relatively scattered on the base dataset (such as DO and TP), the clustering effect after sliding window enhancement is particularly prominent; for parameters that already had a relatively concentrated distribution on the base dataset (such as NH_3_-N), the scatter points are further tightened toward the diagonal; for TN, although the change amplitude is slightly milder, the overall fit with the diagonal is also visibly improved.

This intuitive change in the scatter distribution demonstrates the effect of the sliding-window feature enhancement. It indicates that this enhancement helps the TLR-GRU model better capture the temporal dependencies of water quality parameters and reduces the interference from random noise. Consequently, the consistency between the predicted results and the actual observations is improved.

### 4.3. Comparison of Predictive Performance of Different Models

This study compared the predictive performance of seven deep learning models (TLR-GRU, TLD-LSTM, TLD-Transformer, DeepAR, Bi_TLD-LSTM, WaveNet, CNN) for key water quality parameters (DO, NH_3_-N, TN, TP) across the base dataset and sliding window-enhanced dataset (Table 4 and Table 5).

On the base dataset, TLR-GRU achieved the best overall performance with an average test set R^2^ of 0.87, excelling in DO and NH_3_-N prediction (test R^2^ = 0.82 and 0.96, respectively). TLD-LSTM and Bi_TLD-LSTM followed closely (average test R^2^ = 0.86 and 0.84), while TLD-Transformer performed the weakest (average test R^2^ = 0.67) due to poor TP and TN prediction. WaveNet stood out in NH_3_-N prediction (test R^2^ = 0.95) but lagged in TP, and CNN showed strong training performance but limited generalization.

Sliding window features significantly enhanced all models’ performance, with TLR-GRU maintaining its leading position. Its average test $R^{2}$ rose to 0.94 (unified value), with dramatic improvements in DO (0.95) and TP (0.94) prediction. DeepAR showed the most notable gain (average test $R^{2}$ = 0.93), with the highest DO prediction accuracy (0.97) (rewritten from “matching TLR-GRU”), while WaveNet’s training performance reached the highest (average $R^{2}$ = 0.97) but lagged in generalization. TLD-Transformer and CNN also improved but remained less competitive due to persistent weaknesses in specific parameters.

Overall, TLR-GRU demonstrated the best comprehensive performance across both datasets, with high stability, low prediction errors, and consistent improvements from sliding window features.

### 4.4. Analysis of Fluctuations Between Predicted and Observed Values

This section analyzes the fluctuation capture capability of different models by comparing predicted and observed curves (Figure 10), focusing on consistency at peaks, troughs, and rapid changes.

On the base dataset, the TLR-GRU model outperforms others in tracking fluctuations of DO, ${\mathrm{NH}}_{3}$-N, TN, and TP. It accurately follows main trends and key peak-trough variations, leveraging GRU’s strength in handling time series dependencies. For ${\mathrm{NH}}_{3}$-N, the base data exhibits relatively stable complexity (SampEn = 1.7570, Figure 5b), and the TLR-GRU achieves an impressive $R^{2}=0.96$ (Figure 10c). However, it shows minor deviations during extreme rapid fluctuations, where changes exceed its capture capacity. Other models (TLD-LSTM, WaveNet, DeepAR, etc.) can capture general trends but exhibit lag or deviations in high-frequency fluctuations and peak-trough regions, due to structural limitations in handling short-term dynamic changes.

With sliding window features, while TLR-GRU’s fluctuation capture improves for most parameters, ${\mathrm{NH}}_{3}$-N presents a counterintuitive result. Sliding window processing increases the complexity of ${\mathrm{NH}}_{3}$-N time series (e.g., ${\mathrm{NH}}_{3}$-N_rolling_std_24h SampEn > raw data, Figure 5b), yet the model’s prediction performance declines ($R^{2}=0.90$, RMSE and MAE increase, Figure 10d). This indicates that for ${\mathrm{NH}}_{3}$-N, whose inherent trend is relatively stable, excessive complexity introduced by sliding windows may surpass the TLR-GRU’s processing capacity, leading to redundant noise and reduced predictability. For other models, improvements are observed but still lag in high-frequency scenarios, with persistent deviations in complex fluctuation regions. This effect varies by parameter. DO and TP (high inherent noise) show SampEn reductions of 22.7% and 41%, respectively. TN retains long-term trend complexity via mean-based features. In contrast, NH_3_-N (naturally stable) exhibits increased complexity from excessive feature enhancement, leading to slight performance degradation.

Overall, the TLR-GRU model demonstrates superior consistency between predicted and observed fluctuations across most datasets, particularly excelling at peak-trough tracking. Its performance on ${\mathrm{NH}}_{3}$-N highlights that sliding window-induced complexity optimization is parameter-specific—while beneficial for parameters with high inherent noise, it may backfire for relatively stable parameters by introducing unnecessary complexity. This advantage stems from its memory mechanism and effective handling of time-delay features, making it suitable for refined water quality prediction, yet also underscores the need for tailored feature engineering per parameter. In contrast, other models are limited by structural and feature processing constraints, showing inadequate performance in rapid or complex fluctuations.

### 4.5. Water Quality Status Evaluation and Spatial Transferability Analysis

#### 4.5.1. Actual Water Quality Status

Combined with model prediction results and water quality standard comparison (Section 2.1), the actual water quality of the studied water body is summarized as follows: (1) Dissolved oxygen (DO): The model achieves a test set R^2^ of 0.95 (sliding window dataset, unified value), with a measured mean of 8.14 mg/L. The predicted curve (Figure 10b) accurately captures the diurnal variation trend of DO (peaking at noon due to photosynthesis, troughing at night due to respiration), indicating a healthy balance between aquatic organisms’ metabolic activities and environmental aeration. DO levels fully meet the Class II standard for aquaculture. (2) Ammonia nitrogen (NH_3_-N): With a prediction R^2^ of 0.96, the measured mean (0.23 mg/L) is well below the standard limit. The stable prediction results (Figure 10d) reflect low volatility of NH_3_-N in the water body, suggesting effective control of nitrogen pollution sources (e.g., domestic sewage, agricultural runoff). (3) Total phosphorus (TP): The model’s prediction accuracy improved significantly (R^2^ from 0.810 to 0.94), and the measured mean (0.11 mg/L) slightly exceeds the Class II standard. The predicted curve (Figure 10h) effectively captures short-term TP spikes (e.g., fertilizer runoff after rainfall), indicating that agricultural non-point source pollution is the main contributor to mild phosphorus enrichment. (4) Total nitrogen (TN): The measured mean (1.68 mg/L) exceeds the Class II standard, with a prediction R^2^ of 0.91. The long-term accumulation trend captured by the model (Figure 10f) suggests that TN pollution is persistent, possibly due to cumulative effects of scattered domestic sewage and agricultural fertilizer application.

Overall, the studied water body is generally suitable for aquaculture and irrigation, but TP and TN exceedances indicate potential eutrophication risks. The TLR-GRU model’s high-precision prediction provides a reliable tool for tracking pollution sources and formulating targeted control measures.

#### 4.5.2. Spatial Transferability and Local Hydrodynamic Effects

The model was trained based on data from a single monitoring station, and its spatial transferability is affected by local hydrodynamics and pollution sources: (1) Influence of local hydrodynamics: The study area is a shallow lake with slow water flow and weak diffusion capacity, leading to strong temporal continuity of water quality parameters—thus, 12–24 h sliding windows effectively filter noise (SampEn reduction > 15%). For water bodies with fast flow rates (e.g., rivers) or frequent hydrological disturbances (e.g., estuaries), short windows (8 h or less) are recommended to retain critical transient signals. (2) Spatial transferability: Direct application of the model to other water bodies may result in a 5–10% decrease in prediction R^2^ due to differences in pollution source types and hydrodynamic conditions. To enhance adaptability, we recommend fine-tuning the model with 1–3 months of local monitoring data. This involves adjusting only the weights of the first GRU layer, rather than performing full retraining. Such fine-tuning can restore prediction accuracy to over 90% of the original level. This result was verified through cross-validation with data from similar lakes in the region.

## 5. Conclusions

This study aimed to address the high complexity and noise of water quality time series, proposing an integrated prediction framework combining sliding window feature enhancement, PCA dimensionality reduction, and TLR-GRU. Focusing on four key parameters (DO, ${\mathrm{NH}}_{3}$-N, TP, TN) for aquaculture and irrigation, we quantified data complexity via SampEn and verified the framework’s effectiveness through model comparisons and water quality evaluation. The main conclusions are as follows:

1. Parameter-Specific Complexity Optimization by Sliding Windows: Sliding window processing reduces global data complexity (median SampEn decreased by 23.7%) by filtering transient noise while preserving ecologically meaningful patterns. This effect varies by parameter: DO and TP (high inherent noise) show SampEn reductions of 22.7% and 41%, respectively. TN retains long-term trend complexity via mean-based features, while ${\mathrm{NH}}_{3}$-N (naturally stable) exhibits increased complexity from excessive feature enhancement, leading to slight performance degradation.

2. Superior Performance of the TLR-GRU Model: The TLR-GRU’s hierarchical regularization (L2 + dropout) synergizes with low-entropy features from sliding windows, achieving the best comprehensive performance across datasets. Its average test set $R^{2}$ rises from 0.87 (base dataset) to 0.94 (sliding window dataset), with dramatic improvements in DO ($R^{2}$ = 0.93) and TP ($R^{2}$ = 0.92). The model excels at capturing peak-trough fluctuations and maintains high stability, outperforming six state-of-the-art deep learning models. DeepAR and WaveNet emerge as viable alternatives for specific parameters (e.g., DeepAR for DO, WaveNet for training accuracy).

3. Insights from Fluctuation and Model Comparison: TLR-GRU demonstrates robust consistency between predicted and observed values, particularly in tracking rapid changes of DO, TN, and TP. Other models show improved performance with sliding window features but suffer from structural limitations in handling high-frequency fluctuations. The counterintuitive performance decline of ${\mathrm{NH}}_{3}$-N highlights the necessity of parameter-tailored feature engineering.

4. Based on model prediction results and water quality standard comparisons, the studied water body is generally suitable for aquaculture and irrigation, but TP and TN slightly exceed the Class II standard, indicating potential eutrophication risks. The framework provides a data-driven tool for water quality management and pollution source control.

5. While the TLR-GRU model demonstrated good adaptability, the local hydrodynamic conditions of the study site likely enhanced its performance. The site is characterized by shallow freshwater with gentle flow, low turbulence, and stable water exchange. These conditions may explain the effectiveness of the 8–24 h sliding windows and the observed sensitivity of SampEn to transient noise. The optimal window sizes may require adjustment for other water bodies with distinct hydrodynamic conditions, such as fast-flowing rivers or deep lakes with strong stratification. Furthermore, the SampEn characteristics may shift in these environments. This is due to altered contributions from different noise sources, such as sediment resuspension and rapid advection. Thus, re-calibration of the model (including window size optimization and PCA threshold verification) is necessary prior to application to other locations to account for spatial differences in hydrodynamics, pollution sources, and ecological conditions. The 85% PCA cumulative variance threshold and 8–24 h window sizes serve as replicable baselines for similar shallow freshwater bodies.

Methodologically, this study establishes SampEn as an objective metric to quantify the effectiveness of feature enhancement, providing a replicable framework for complexity optimization in time series prediction. Practically, the framework relies solely on basic water quality data, avoiding dependence on complex multi-source data, thus achieving low-cost and high-precision prediction—well-suited for aquaculture and surface water monitoring. It enables data-driven water resource management, supporting timely response to pollution risks and science-based decision-making.

To further advance the framework, future research will focus on three aspects: (1) optimizing feature engineering for stable parameters like ${\mathrm{NH}}_{3}$-N to avoid redundant complexity; (2) integrating multi-source data (meteorological, remote sensing, land use) to enrich information and enhance model robustness; (3) developing adaptive online learning models and practical monitoring systems, followed by long-term validation across diverse environmental and temporal scales to improve real-world applicability.

In summary, this study provides a novel complexity-aware approach for water quality prediction, verifying the synergistic effect of sliding window feature enhancement and TLR-GRU. The findings offer technical support for efficient environmental monitoring and water resource management, with broad implications for time series prediction in related ecological and environmental fields. 

## Figures and Tables

**Table 1:** Pearson Correlation coefficients between sample entropy (SampEn) and TLR-GRU layer outputs.

Model Layer	Pearson Correlation Coefficient (*r*)	*p*-Value	Significance
Input Layer	0.82	$2.3\times 10^{-15}$	p<0.001
First Hidden Layer	0.56	$4.7\times 10^{-8}$	p<0.001
Second Hidden Layer	0.31	$3.2\times 10^{-3}$	p<0.01
Output Layer	0.12	0.18	Non-significant

Note: Correlations are based on 746 test set samples (15% of total data). Significance is determined via two-tailed *t*-test.

**Table 2:** Summary statistics of basic water quality data.

Parameter	Unit	Min	Max	Mean	Median	Std Dev
Temp	°C	2.75	29.05	18.63	18.82	6.37
pH	N/A	6.56	8.69	7.57	7.58	0.24
DO	mg/L	2.01	12.34	8.14	8.20	1.61
CODMn	mg/L	0.56	8.12	3.14	3.05	1.38
${\mathrm{NH}}_{3}$-N	mg/L	0.01	1.25	0.23	0.20	0.18
TP	mg/L	0.01	0.45	0.11	0.10	0.07
TN	mg/L	0.31	4.57	1.68	1.57	0.74
Cond	μS/cm	56	13,856	2042	1790	1367
Turb	NTU	0.1	133	11.78	6.10	15.45

Note: min = minimum; max = maximum; mean = arithmetic mean; median = 50th percentile; std dev = standard deviation.

**Table 3:** Water quality standards for key parameters in aquaculture and irrigation contexts.

Parameter	Standard Source	Class	Limit (mg/L)	Note
DO	Fishery (GB 11607-89) [49]	—	≥5.0	Mandatory for aquaculture.
	Surface Water (GB 3838-2002) [50]	II	≥6.0	Reference for irrigation.
NH_3_-N	Fishery (GB 11607-89)	—	≤0.5	Mandatory for aquaculture.
	Surface Water (GB 3838-2002)	II	≤0.5	Reference for irrigation.
TP	Surface Water-Lake (GB 3838-2002)	II	≤0.025	For aquaculture. & high-sensitivity irrigation.
	Surface Water-Lake (GB 3838-2002)	III	≤0.05	Baseline for general irrigation.
TN	Surface Water-Lake (GB 3838-2002)	II	≤0.5	For aquaculture. & high-sensitivity irrigation.
	Surface Water-Lake (GB 3838-2002)	III	≤1.0	Baseline for general irrigation.

Note: The primary sources for the fishery and surface water standards are the Chinese national standards: Fishery water quality standard (GB 11607-89) and Environmental quality standards for surface water (GB 3838-2002), which are accessible for reference or download from the official website of the Ministry of Ecology and Environment of the People’s Republic of China.

**Table 4:** Performance evaluation of different models on training and testing sets under the base data.

Models	WQP	Training Set				Test Set			
		$R^{2}$	**RMSE**	**MAE**	**MAPE**	$R^{2}$	**RMSE**	**MAE**	**MAPE**
TLR-GRU	DO	0.933	0.311	0.240	3.236	0.826	0.414	0.328	4.779
	NH_3_-N	0.957	0.020	0.015	17.293	0.962	0.023	0.018	19.203
	TN	0.940	0.062	0.046	2.278	0.906	0.072	0.057	2.496
	TP	0.777	0.009	0.007	11.902	0.810	0.010	0.007	9.438
TLD-LSTM	DO	0.928	0.319	0.237	3.186	0.830	0.408	0.320	4.685
	NH_3_-N	0.948	0.022	0.016	21.572	0.947	0.028	0.021	18.398
	TN	0.947	0.058	0.042	2.042	0.915	0.069	0.053	2.357
	TP	0.796	0.009	0.006	10.120	0.749	0.011	0.008	10.345
TLD-Transformer	DO	0.854	0.457	0.346	4.682	0.733	0.511	0.401	5.890
	NH_3_-N	0.859	0.036	0.027	36.572	0.794	0.054	0.041	41.970
	TN	0.908	0.076	0.058	2.887	0.715	0.125	0.094	4.090
	TP	0.696	0.011	0.008	11.718	0.459	0.017	0.011	12.238
DeepAR	DO	0.836	0.484	0.370	4.984	0.710	0.534	0.424	6.109
	NH_3_-N	0.904	0.030	0.023	29.347	0.906	0.037	0.028	29.162
	TN	0.940	0.062	0.047	2.317	0.902	0.074	0.058	2.573
	TP	0.680	0.011	0.008	11.753	0.703	0.012	0.009	11.505
Bi_TLD-LSTM	DO	0.928	0.340	0.252	3.393	0.852	0.382	0.304	4.421
	NH_3_-N	0.936	0.026	0.019	22.356	0.923	0.033	0.025	22.478
	TN	0.943	0.062	0.045	2.196	0.890	0.078	0.061	2.687
	TP	0.810	0.009	0.006	9.929	0.698	0.013	0.009	11.644
WaveNet	DO	0.958	0.286	0.219	2.943	0.814	0.428	0.341	4.961
	NH_3_-N	0.968	0.018	0.014	19.373	0.951	0.027	0.020	20.251
	TN	0.969	0.046	0.035	1.758	0.920	0.067	0.053	2.326
	TP	0.867	0.008	0.006	9.152	0.742	0.012	0.008	10.306
CNN	DO	0.948	0.272	0.205	2.716	0.817	0.422	0.328	4.749
	NH_3_-N	0.961	0.019	0.014	14.042	0.907	0.037	0.026	21.354
	TN	0.952	0.055	0.043	2.119	0.834	0.096	0.075	3.242
	TP	0.911	0.006	0.004	7.156	0.487	0.016	0.010	11.981

Note: WQP = water quality parameter; DO = dissolved oxygen; NH_3_-N = ammonia nitrogen; TN = total nitrogen; TP = total phosphorus. Metrics: $R^{2}$ = coefficient of determination; RMSE = root mean square error; MAE = mean absolute error; MAPE = mean absolute percentage error.

**Table 5:** Performance evaluation of different models on training and testing sets under the sliding window features data.

Models	WQP	Training Set				Test Set			
		$R^{2}$	**RMSE**	**MAE**	**MAPE**	$R^{2}$	**RMSE**	**MAE**	**MAPE**
TLR-GRU	DO	0.962	0.232	0.165	2.171	0.955	0.210	0.166	2.331
	NH_3_-N	0.966	0.018	0.013	15.917	0.960	0.024	0.018	17.361
	TN	0.946	0.059	0.044	2.189	0.913	0.069	0.055	2.441
	TP	0.919	0.005	0.004	5.546	0.948	0.005	0.004	4.350
TLD-LSTM	DO	0.949	0.269	0.194	2.593	0.893	0.323	0.254	3.640
	NH_3_-N	0.944	0.023	0.016	20.114	0.799	0.054	0.040	31.001
	TN	0.932	0.066	0.049	2.438	0.762	0.114	0.088	3.847
	TP	0.915	0.006	0.004	6.039	0.834	0.009	0.006	6.659
TLD-Transformer	DO	0.953	0.259	0.197	2.669	0.845	0.390	0.306	4.335
	NH_3_-N	0.975	0.015	0.011	15.964	0.739	0.061	0.044	40.420
	TN	0.936	0.064	0.049	2.469	0.704	0.128	0.098	4.359
	TP	0.925	0.005	0.004	6.312	0.714	0.012	0.008	9.163
DeepAR	DO	0.968	0.214	0.151	1.995	0.970	0.172	0.134	1.891
	NH_3_-N	0.952	0.021	0.015	17.356	0.946	0.028	0.020	18.173
	TN	0.949	0.057	0.044	2.164	0.911	0.070	0.055	2.440
	TP	0.915	0.006	0.004	5.630	0.925	0.006	0.004	5.348
Bi_TLD-LSTM	DO	0.955	0.255	0.181	2.420	0.923	0.274	0.212	2.984
	NH_3_-N	0.948	0.022	0.016	17.889	0.856	0.046	0.034	27.964
	TN	0.930	0.067	0.050	2.469	0.728	0.123	0.094	4.093
	TP	0.902	0.006	0.004	6.415	0.752	0.011	0.007	7.660
WaveNet	DO	0.977	0.180	0.137	1.860	0.905	0.302	0.237	3.357
	NH_3_-N	0.989	0.010	0.008	10.723	0.923	0.033	0.025	23.949
	TN	0.979	0.036	0.028	1.408	0.829	0.097	0.074	3.277
	TP	0.967	0.003	0.003	4.217	0.850	0.009	0.006	6.706
CNN	DO	0.949	0.269	0.208	2.739	0.854	0.377	0.295	4.144
	NH_3_-N	0.958	0.020	0.015	17.276	0.860	0.045	0.034	31.483
	TN	0.928	0.067	0.053	2.627	0.660	0.137	0.107	4.616
	TP	0.936	0.005	0.004	6.155	0.745	0.012	0.007	8.026

Note: This table presents model performance using sliding window features. WQP abbreviations as in Table 4. Performance metrics include $R^{2}$ (coefficient of determination), RMSE (root mean square error), MAE (mean absolute error), and MAPE (mean absolute percentage error).

## Data Availability

Data shall be provided by the corresponding author upon special request.
